# Behavioral Changes as the Initial Presentation of Primary Central Nervous System Lymphoma: A Case Report

**DOI:** 10.7759/cureus.99146

**Published:** 2025-12-13

**Authors:** Bishoy Manqaryos, Michael Shakhloul, Ahmed Amer

**Affiliations:** 1 Emergency Department, Royal Surrey County Hospital, Guildford, GBR; 2 Emergency Medicine, Egyptian Medical Syndicate, Cairo, EGY

**Keywords:** behavior changes, brain mass, brain tumors, frontal lobe mass, large hemi-cranial mass

## Abstract

Brain tumors typically present with headaches, nausea, vomiting, seizures, or focal neurological deficits. However, neuropsychiatric manifestations, such as personality change and behavioral disturbance, may be the earliest signs, particularly when the frontal or temporal lobes are involved. We describe a 69-year-old man who presented repeatedly to his general practitioner (GP) with progressive behavioral changes and intermittent headaches. Initial investigations were unremarkable, and he received cognitive-behavioral therapy without improvement. He later developed facial deviation and limb numbness. Computed tomography (CT) of the head revealed a large, right-sided, space-occupying lesion, subsequently confirmed by magnetic resonance imaging (MRI) to be an intracranial tumor. Histopathology following biopsy confirmed primary central nervous system lymphoma (PCNSL). This case highlights the importance of considering intracranial pathology in patients with persistent or unexplained behavioral changes and underscores the role of early neuroimaging in preventing diagnostic delays.

## Introduction

Brain tumors encompass a heterogeneous group of intracranial neoplasms originating from neural tissue, meninges, lymphatic cells, or metastatic deposits. They are broadly classified as primary tumors, arising from intracranial tissues, and secondary tumors, which originate from systemic malignancies such as lung, breast, and melanoma. Primary brain tumors are further categorized according to histogenesis and malignancy grade into gliomas, meningiomas, schwannomas, pituitary adenomas, and primary central nervous system lymphoma (PCNSL) [1-3].

PCNSL is a rare and aggressive form of extranodal non-Hodgkin’s lymphoma confined to the brain, leptomeninges, spinal cord, or eyes in the absence of systemic disease. It most commonly affects older adults and is classically associated with deep periventricular lesions, although frontal lobe involvement is increasingly recognized [2].

Clinical manifestations of brain tumors vary depending on anatomical location, growth rate, and associated edema or mass effect. Classic symptoms include headache, nausea, vomiting, seizures, and focal neurological deficits [1]. However, tumors involving the frontal and temporal lobes may initially present with predominantly psychiatric or cognitive symptoms such as apathy, personality change, emotional lability, impaired judgment, executive dysfunction, or psychosis [4-6].

Such presentations create significant diagnostic difficulty, as the differential diagnosis is extensive and includes major depressive disorder, late-onset psychosis, frontotemporal dementia, delirium, and medication-induced psychiatric syndromes. In primary care settings, these symptoms are frequently attributed to functional psychiatric disorders or age-related cognitive decline, leading to delayed neuroimaging and diagnosis [4-6].

This report describes a patient whose insidious behavioral changes preceded focal neurological deficits and culminated in the diagnosis of PCNSL. The case emphasizes the importance of clinical vigilance and early neuroimaging in patients presenting with atypical or persistent neuropsychiatric symptoms.

## Case presentation

A 69-year-old man presented multiple times to his general practitioner (GP) because of progressive behavioral changes over several months. His family reported increasing irritability and difficulty organising tasks such as leaving the kitchen in disarray. He also experienced intermittent headaches that were not clearly localised. This continued for about five weeks. Initial blood tests were normal, and he was referred for cognitive-behavioral therapy (CBT). Despite attending sessions, his behavioral symptoms continued to worsen.

Approximately one week prior to his final GP visit, he developed left-sided facial weakness, numbness affecting the left upper limb, and progressive deviation of the mouth. On the day of his emergency department (ED) presentation, he was noted to have new-onset left eyelid drooping and appeared increasingly anxious and withdrawn.

In the ED, he was alert and oriented but displayed mild disinhibition. Cranial nerve examination revealed left facial asymmetry and ptosis. Mild sensory reduction was noted in the left upper limb. The remainder of the neurological examination was non-focal.

**Figure 1:**
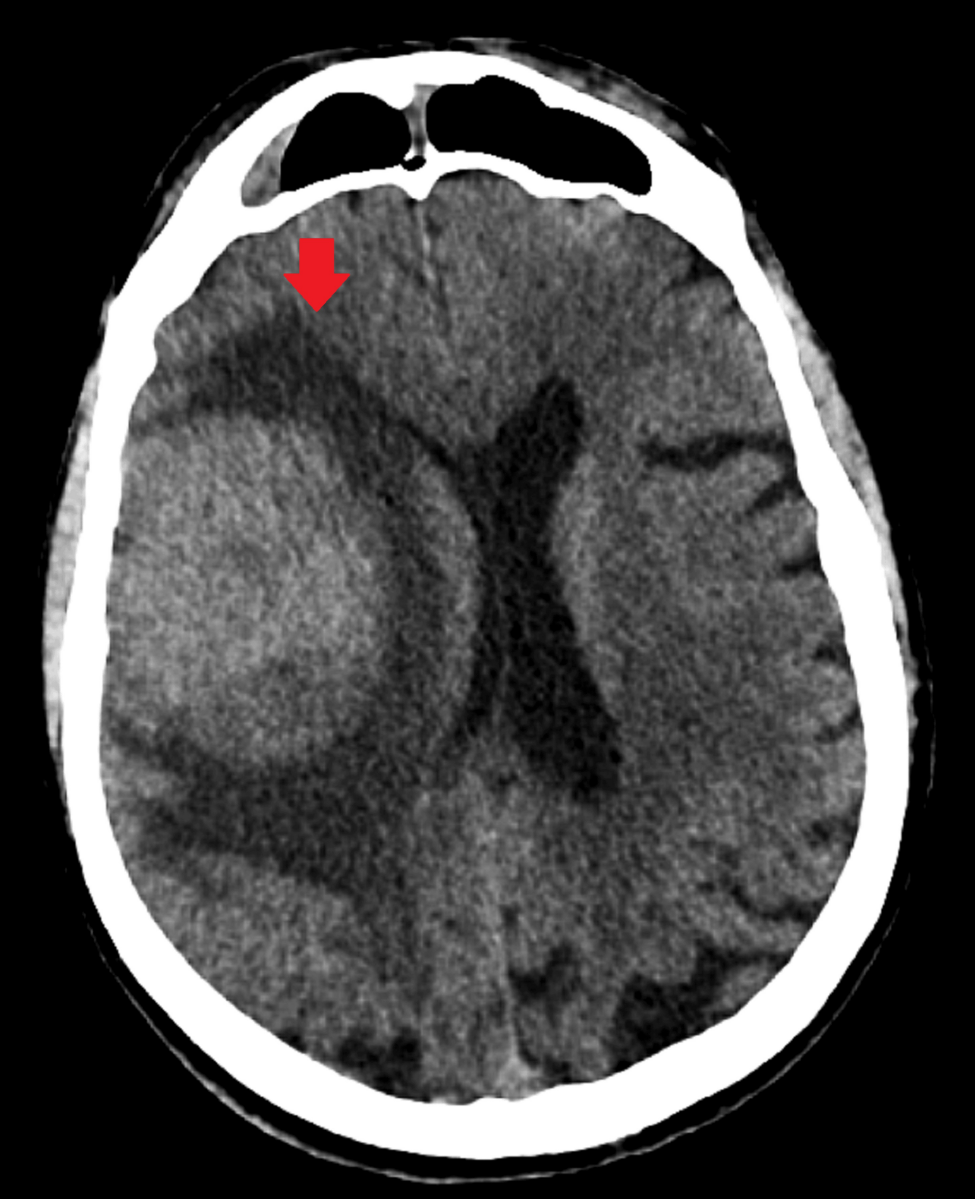
Transverse section of CT of the brain shows a brain mass

**Figure 2:**
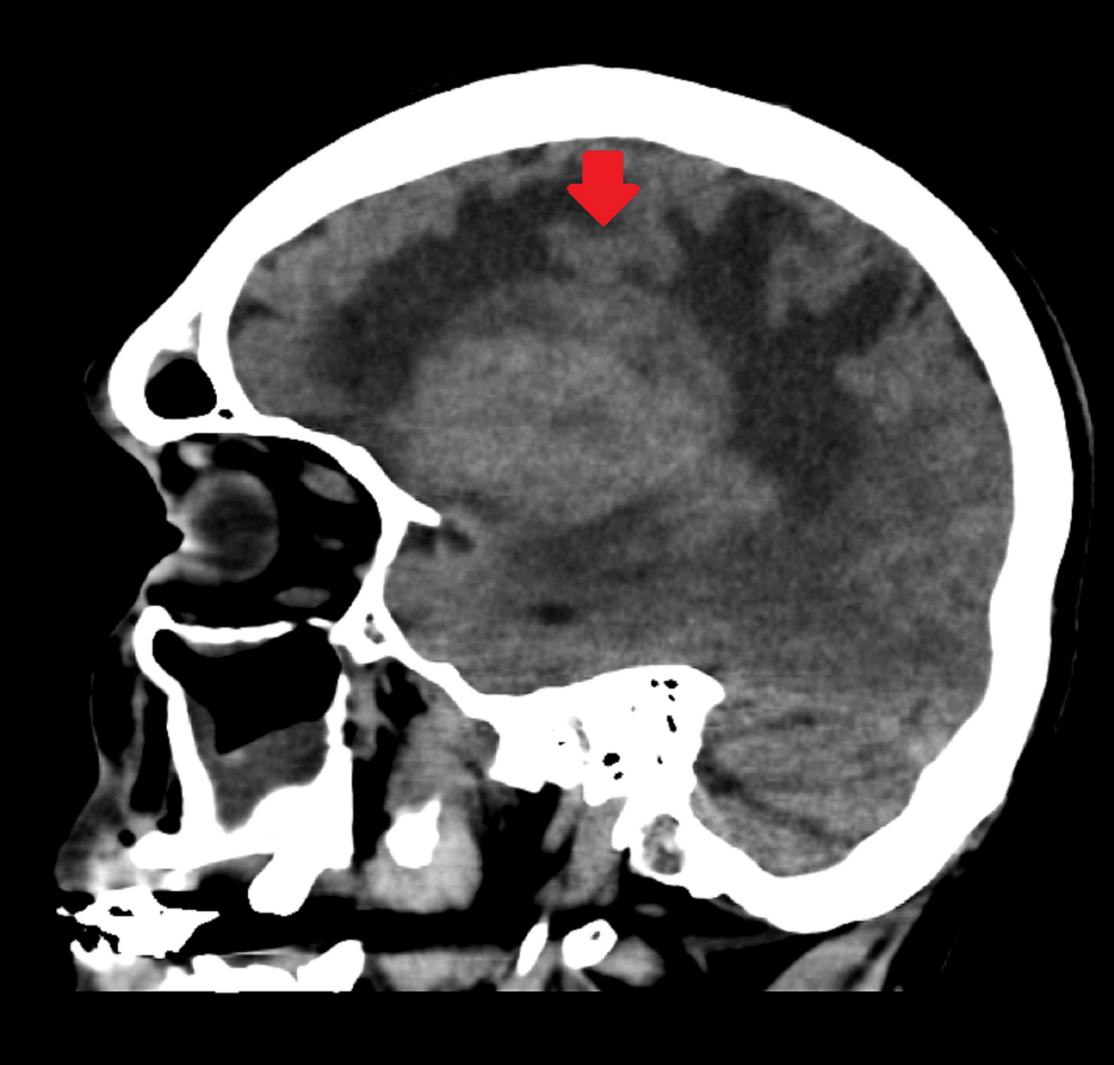
Sagittal section of CT of the brain shows a brain mass

**Figure 3:**
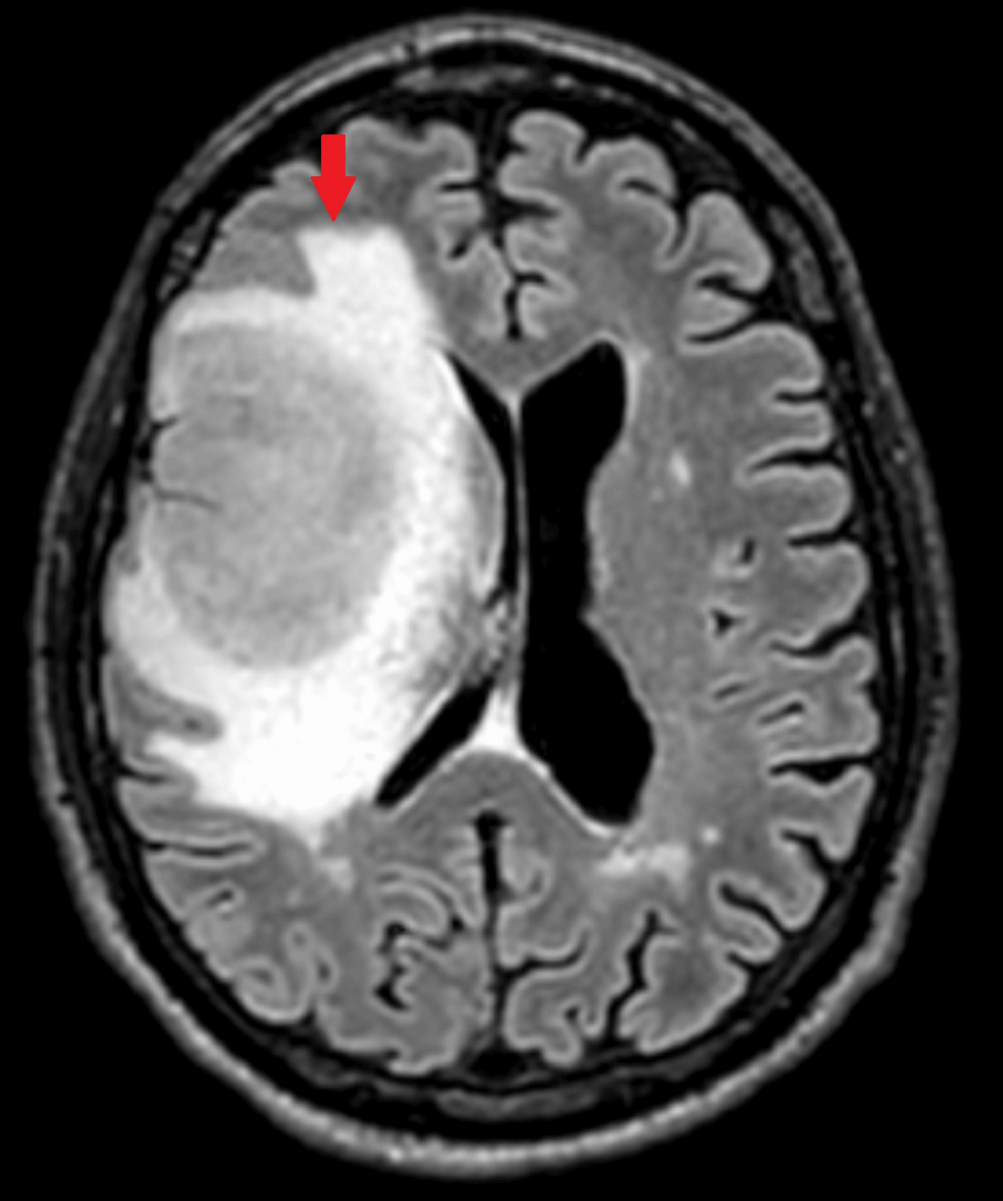
Transverse section of MRI shows a brain mass

**Figure 4:**
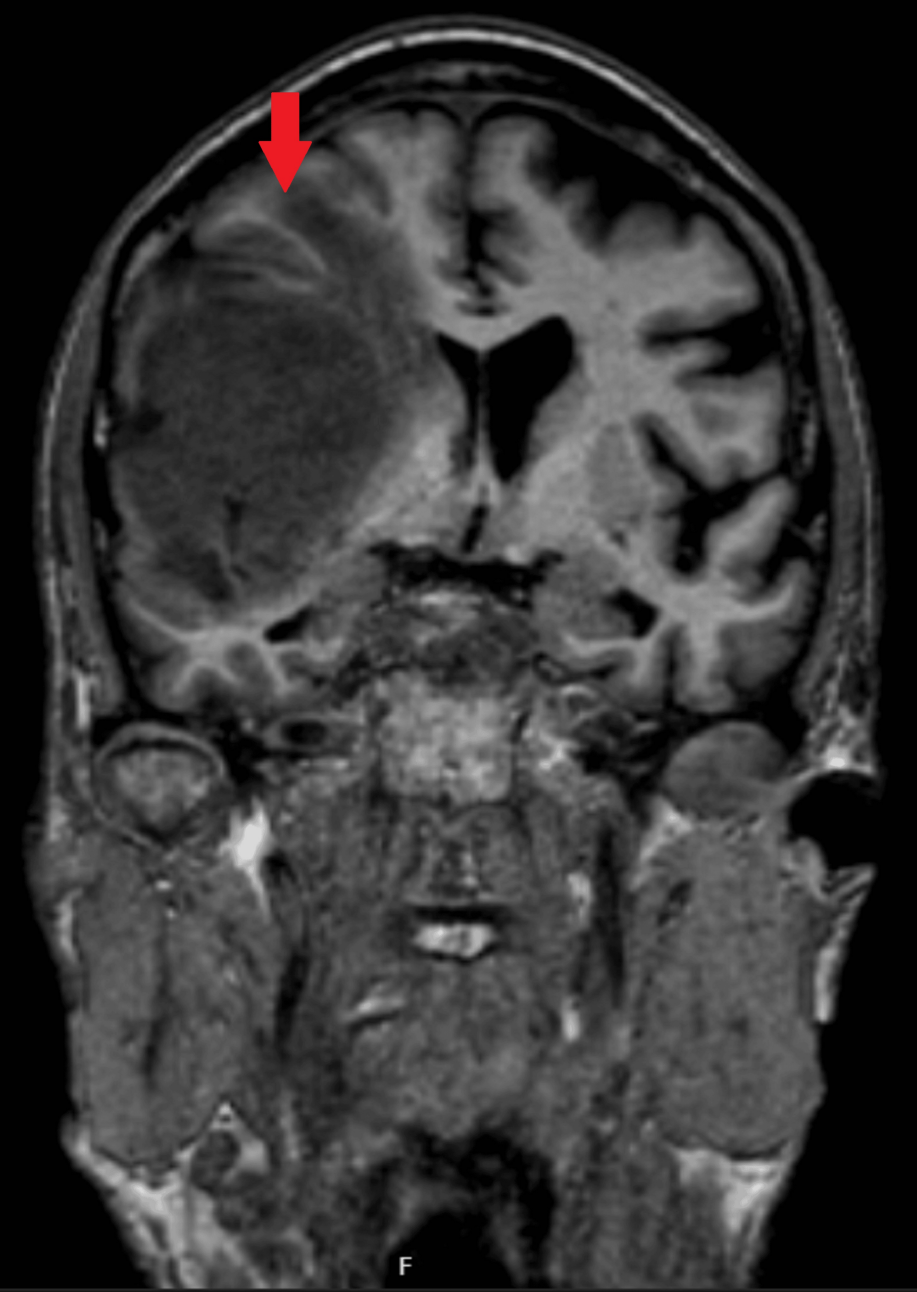
Coronal section of MRI shows a brain mass

A non-contrast CT head scan demonstrated a large right frontal mass with surrounding vasogenic edema and radiological features of raised intracranial pressure (Figures 1, 2). He was referred urgently via the two-week suspected cancer pathway. Magnetic resonance imaging (MRI) subsequently confirmed a right frontal space-occupying lesion consistent with a primary intracranial neoplasm (Figures 3, 4).

The patient was admitted under the acute medicine team for further neurological evaluation. Steroids were initiated, and an urgent multidisciplinary team (MDT) referral was made. Following the MDT discussion, a brain biopsy was recommended. An uncomplicated right frontal lobe biopsy was performed, and histology confirmed a malignant tumour consistent with PCNSL.

A positron emission tomography (PET) scan subsequently revealed a lung mass. Bronchoscopy confirmed operable lung adenocarcinoma, which was successfully resected. The patient has since undergone chemotherapy and autologous stem cell transplantation and is now under regular follow-up.

## Discussion

Brain tumors exhibit a wide spectrum of neurological and neuropsychiatric manifestations, largely influenced by their anatomical location, size, and rate of growth. Lesions affecting the frontal and temporal lobes are particularly notorious for producing behavioral and cognitive changes that may precede overt neurological deficits. Frontal lobe pathology can disrupt executive function, emotional regulation, planning, judgment, and social behavior, often resulting in irritability, apathy, disinhibition, or subtle personality change. These symptoms can closely mimic primary psychiatric disorders and are well-documented sources of diagnostic delay in neuro-oncology [1,4-6]. A systematic review by Moroşan et al. systematically examined the role of neuropsychiatric symptoms as early indicators of intracranial tumours. This review identified that these symptoms may arise prior to overt neurological deficits, and argued that their presence should prompt a lower threshold for neuro-imaging in appropriate clinical contexts [4].

In many patients, early behavioral manifestations are misattributed to psychological or social stressors, especially when classic symptoms, such as persistent morning headaches, seizures, or clear lateralizing neurological signs, are absent. Our patient’s initial presentation was dominated by irritability, inappropriate comments, and reduced ability to perform previously routine tasks-symptoms that led clinicians to pursue a psychological explanation and initiate cognitive-behavioral therapy. This aligns with findings in the literature that frontal lobe tumors often produce insidious personality changes that are easily misinterpreted as depression, stress reactions, functional disorders, or early dementia [5].

The challenge lies in distinguishing psychiatric presentations caused by intracranial pathology from primary psychiatric illness. Red flags include progressive symptom evolution, lack of therapeutic response, and the emergence of even subtle neurological findings. In this case, the patient’s gradual development of facial asymmetry, left upper limb numbness, and ptosis shifted the clinical suspicion toward an organic etiology. These focal signs are consistent with the involvement of the right frontal region and adjacent motor pathways, where mass effect or vasogenic edema may produce contralateral weakness or sensory alteration [7-13].

Neuroimaging remains the cornerstone for diagnosing intracranial tumors. While MRI provides superior detection, tissue characterization, and anatomical detail, CT is often the first-line modality in the emergency setting because of its rapidity and availability [7]. In this patient, the CT scan revealed a large right frontal space-occupying lesion with surrounding edema, prompting urgent MRI confirmation. The radiological appearance of vasogenic edema, mass effect, and contrast enhancement patterns are critical in differentiating high-grade gliomas from metastases or benign lesions [7].

The diagnostic pathway in this case underscores several well-recognized challenges in clinical neuropsychiatry. First, brain tumors may present with nonspecific psychological or cognitive symptoms, causing prolonged intervals between symptom onset and diagnosis. Second, reliance on psychological explanations without consideration of organic causes can delay the use of neuroimaging, which is essential for early detection. Third, the literature consistently demonstrates that prognosis in high-grade brain tumors correlates with early diagnosis and timely intervention [4]. While early imaging is not warranted for every behavioral complaint, persistent or progressive symptoms, particularly when lacking a clear psychiatric basis, should prompt consideration of structural pathology.

Our case also aligns with a systematic review by Ghandour et al., which found that brain tumours may initially present with isolated psychiatric or behavioural symptoms, often months before neurological signs appear. The review highlighted that such early manifestations frequently lead to diagnostic delay, as they are easily misattributed to functional or psychiatric causes [8]. Our patient’s presentation reflects this pattern, reinforcing the importance of maintaining suspicion for intracranial pathology when behavioural change is unexplained or disproportionate, in order to avoid delayed diagnosis. 

Seddighi et al. also reviewed neuropsychological disorders in brain tumor patients, reporting that psychiatric symptoms such as anxiousness, obsession, and depression were prevalent even before neurological signs appeared. The review stressed the importance of neuroimaging in patients presenting with atypical mental status changes, even in the absence of classic focal findings [9].

A 2025 case series and discussion, summarized by Srivastav et al., referenced large meta-analyses indicating that brain tumor patients may experience neuropsychiatric symptoms, with some of them presenting primarily with psychiatric complaints, especially with frontal and temporal lobe tumors. These cases can be misdiagnosed as primary psychiatric illness, causing further diagnostic delay [10].

National Institute for Health and Care Excellence (NICE) guidelines emphasize the importance of recognising unusual, progressive, or unexplained neurological symptoms and considering early imaging when red-flag features are present [5,11]. Behavioral changes that evolve over months, coexist with subtle neurological abnormalities, or fail to respond to psychological therapy fall into this category [12,13].

PCNSL is uniquely associated with neuropsychiatric presentations, owing to its predilection for deep frontal and periventricular structures. Unlike primary gliomas, PCNSL fragments corticostriatal circuits and limbic pathways, leading to early personality changes, executive dysfunction, and behavioral dysregulation [14]. This tumor type is also frequently misdiagnosed because imaging features may initially resemble high-grade gliomas or metastases [15].

Our case demonstrates the need for clinicians to adopt an integrative diagnostic approach when evaluating patients with behavioral disturbances. A holistic assessment incorporating neurological examination, collateral history from family or caregivers, and timely neuroimaging is essential to avoid diagnostic delay. Recognizing that behavioral or personality changes may represent early manifestations of brain tumors can lead to earlier diagnosis, more effective treatment, and improved patient outcomes.

## Conclusions

This case illustrates that brain tumors may present initially with behavioral changes that resemble psychiatric disorders, particularly when involving the frontal lobes. Persistent or worsening neuropsychiatric symptoms, especially when accompanied by subtle neurological abnormalities, should raise suspicion for intracranial pathology. Early neuroimaging is critical to avoid diagnostic delay, guide appropriate management, and improve patient outcomes. Clinicians should maintain a broad differential diagnosis when assessing behavioral changes of unclear origin.
